# Variation in Vegetation and Its Driving Force in the Pearl River Delta Region of China

**DOI:** 10.3390/ijerph191610343

**Published:** 2022-08-19

**Authors:** Shulin Chen, Zhenghao Zhu, Xiaotong Liu, Li Yang

**Affiliations:** College of Economics and Management, Nanjing Forestry University, Nanjing 210000, China

**Keywords:** NDVI, driving factors, climatic factors, human activities, residual analysis

## Abstract

Vegetation is an important part of a regional ecological environment and vegetation coverage can reflect the health of a regional ecological environment. Through an analysis of and research into changes in the vegetation NDVI (normalized difference vegetation index) and its driving factors in the Pearl River Delta region, the spatial–temporal pattern of vegetation changes and the driving factors can be measured. It is of significance to improve the ecological environment quality of the Pearl River Delta region and to promote the sustainable development of the regional economy. Based on SPOT/VEGETATION NDVI satellite remote sensing data, meteorological data, population density data, and gross domestic product (GDP) data during the period 2000–2019, this paper analyzed the temporal and spatial trends of the vegetation NDVI as well as the climate factors and human activities in the Pearl River Delta on a pixel scale. The correlations between the vegetation NDVI and precipitation, temperature, population density, GDP, and other factors were also estimated. The results showed that during the period 2000–2019, the annual mean NDVI significantly increased, with a growth rate of 0.0044 (*R*^2^ = 0.71, *p* < 0.0001). The NDVI in the center of the Pearl River Delta was lower than that in other regions. As far as the driving factors of the NDVI were concerned, among the climatic factors, the response of the NDVI to temperature was higher than that for precipitation in the Pearl River Delta. Human activities had changed from a negative hindering effect on the NDVI to a positive promoting effect. The correlation between the NDVI and the GDP was higher than that for population density. Policy factors such as the “Grain for Green Project” as well as an increase in the sown area of crops and land use changes were also important driving factors of the NDVI. It is suggested that the NDVI can be increased by the implementation of artificial afforestation policies, building a “Green City”, and moderately increasing the sown area of crops.

## 1. Introduction

Vegetation is an important part of terrestrial ecosystems; it is the link between the soil, water, and the atmosphere [1,2] and it plays an indicative role in global environmental changes. Vegetation coverage can reflect the composition of vegetation types on the surface and the growth state of vegetation as well as reflecting the health status of regional ecosystems. The normalized difference vegetation index (NDVI) can reflect regional vegetation coverage; it has been widely used to reflect the spatial patterns of vegetation coverage [3,4].

The driving factors affecting NDVI changes can be divided into climatic factors and human factors. On the one hand, climate change can affect the growth of vegetation; on the other hand, human activities can also have positive and negative impacts on the vegetation cover. Previous studies have pointed out that there is a significant correlation between the NDVI and climatic factors; temperature and precipitation were generally considered to be the main drivers of global vegetation growth [5,6]. On a global scale, several studies have shown that the NDVI is significantly positively correlated with temperature in the middle and high latitudes of the Northern Hemisphere [7]. In terrestrial ecosystems, the availability of water resources limits the growth and production of plants [8]; the correlation between the vegetation NDVI and precipitation is particularly significant in arid and semi-arid regions [9]. However, at a regional scale, vegetation growth characteristics mainly depend on the spatial heterogeneity of climatic and topographical factors [10,11]. In China, the regions where the NDVI is highly sensitive to climatic factors are located at higher altitudes [12] such as the mountain grasslands and shrubs in southeastern Tibet and the temperate broad-leaved forests and mixed forests in Northeast China [13,14]. Studies have shown that natural vegetation in forests, wetlands, and alpine tundra regions are highly sensitive to temperature changes, especially small, spatially dispersed areas [15]. However, in Northwest China and the subtropical and tropical regions, precipitation changes have had a relatively large contribution to vegetation dynamics [12].

In addition to climatic factors, human activities also affect the vegetation NDVI [16] and, in the short term, human activities can accelerate the rate of vegetation change [17]. Previous studies have pointed out that the land use changes caused by human activities such as global farmland reclamation and urbanization have become an important factor affecting the spatial distribution of vegetation [18]. Over the past few decades, excessive deforestation and the expansion of cropland and urban land worldwide has led to land degradation, desertification, and soil erosion, which in turn has resulted in a reduction in the regional vegetation NDVI [19,20,21]. There are also a few studies showing that in most parts of the world, the population density and GDP have a significant impact on vegetation NDVI changes [22].

With extensive human activities and the neglect of the ecological environment, ecological problems such as land degradation, desertification, and soil erosion have gradually appeared in a few areas of China [19,20,21]. China has implemented a series of ecological protection policies such as the “Grain for Green Project” since 1999 [23]. Therefore, research into the impact of human activities on the NDVI has become increasingly significant [24]. However, the NDVI is affected by both climatic factors and human factors. How to separate the climatic factors and quantify the impact of human activities on the NDVI is one of the hot issues faced by ecologists. The RESTREND (residual trend analysis) model can calculate the difference between the predicted NDVI and the observed NDVI to separate the NDVI dominated by climatic factors and obtain the NDVI residual. The residual analysis results are considered to be the human-factor-dominated NDVI; these have been widely used all over the world [25,26].

The Pearl River Delta Economic Zone is a pioneering area of China regarding reform and opening up; it is the leader that radiates and drives the development of South China, Central China, and Southwest China. Together with the Yangtze River Delta and Beijing-Tianjin-Hebei, it has become one of the three largest urban agglomerations in China with the largest population density and the strongest comprehensive strength [27]. With a rapid increase in the population density and a rapid improvement to the urbanization level, the contradiction between human activities and regional vegetation coverage has become more severe and significant. Studying the spatial–temporal patterns and driving factors of the vegetation NDVI in the Pearl River Delta has certain reference and guiding values for future land use planning, afforestation policies, and crop cultivation area planning in the Pearl River Delta.

To date, previous studies have focused on the impact of natural factors and land use types on the NDVI in the Pearl River Delta. Several studies have shown that the impact of temperature and precipitation on the NDVI in the Pearl River Delta region have the characteristics of different time lags [28,29]; the effect of temperature on the NDVI was stronger than that of precipitation [29]. The NDVI in the Pearl River Delta region has the characteristics of high spatial agglomeration and, due to the influence of the topography and urban distribution, the distribution patterns of low-vegetation agglomeration and high-vegetation agglomeration areas were significant and stable. The land use change also affected the NDVI; the conversion of cropland and forest to urban land was the main cause of vegetation cover degradation [30]. However, few studies have analyzed the response of the NDVI to human factors on a pixel scale. Therefore, studying the driving effect of human activities on the NDVI in the Pearl River Delta on a pixel scale is helpful for the government to plan rational land use and formulate vegetation restoration policies.

Given the above scientific challenges, in this study we investigated the impact of human activities such as the GDP and population density on the NDVI on a pixel scale. The objectives of this study were to: (1) analyze the temporal and spatial changes of the NDVI in the Pearl River Delta; (2) analyze the impact of climatic factors such as precipitation and temperature on the vegetation NDVI; and (3) analyze the impact of human activities such as the GDP, population density, and policy on the NDVI.

## 2. Materials and Methods

### 2.1. Study Area

**Figure 1:**
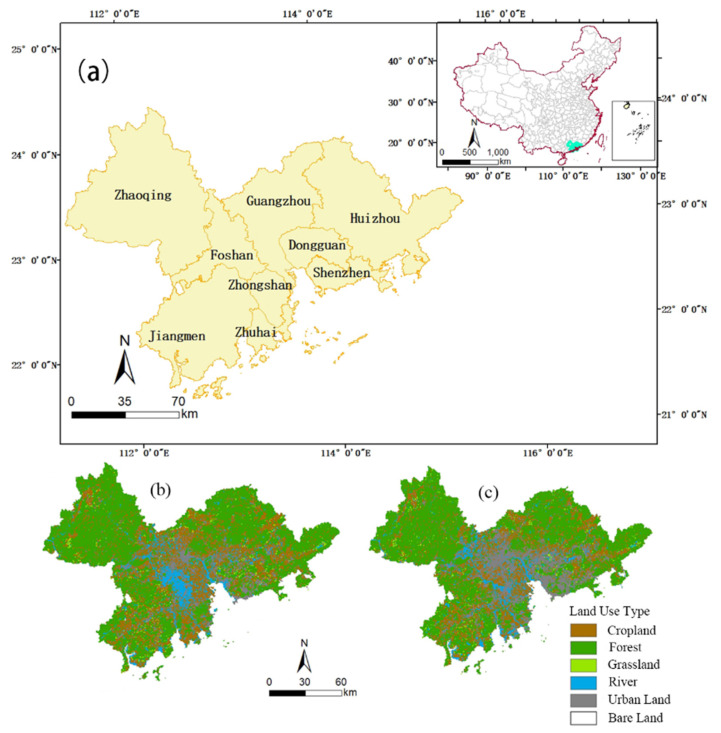
(**a**) Location of the Pearl River Delta Region and the land use type in (**b**) 2000 and (**c**) 2020.

The Pearl River Delta is located at 112°45′–113°50′ E (longitude) and 21°31′–23°10′ N (latitude) (see Figure 1a). It is a composite delta formed by sediment brought by the Pearl River to the mouth of the Pearl River. The Pearl River Delta includes nine cities (Guangzhou, Foshan, Zhaoqing, Shenzhen, Dongguan, Huizhou, Zhuhai, Zhongshan, and Jiangmen) covering an area of 56,000 square kilometers. Most of the Pearl River Delta is located in the south subtropical zone and has a subtropical marine monsoon climate. The annual average temperature is 21.4–22.4 °C and the annual average precipitation is 1600–2300 mm. The Pearl River Delta became an open coastal economic zone in 1986 and is one of the fastest growing regions in China. From 2000 to 2019, the GDP of the Pearl River Delta increased year by year, showing a rapid growth trend overall. Its GDP increased from 0.16 trillion USD in 2000 to 1.28 trillion USD in 2019. According to the land use maps in the years 2000 and 2020 (see Figure 1b,c), the cropland decreased by 2385.78 km^2^ and was mainly converted into forest and urban land. The forest land decreased by 948.45 km^2^ and was mainly converted into cropland and urban land. The grassland decreased by 13.93 km^2^ and was mainly converted into forest land. The area of the river decreased by about 509.55 km^2^ and was mainly converted into cropland and urban land. A significant amount of the cropland, forest land, and river were converted into urban land; therefore, the urban land increased by 3878.63 km^2^. Bare land decreased by 19.87 km^2^ and was mainly converted into forest land, urban land, and grassland.

### 2.2. Data Sources and Preprocessing

The datasets we used in this paper were the NDVI, meteorological datasets, the annual increment of artificial afforestation, the sown area of crops, land use maps, the GDP, and the population density in the Pearl River Delta from 2000 to 2019. The SPOT/VEGETATION NDVI, land use types, GDP, and population density were downloaded from the Resource and Environmental Science and Data Center of the Chinese Academy of Sciences (http://www.resdc.cn/Default.aspx, accessed on 25 April 2022). We estimated the 12 month mean NDVI to obtain the annual NDVI for each year. Based on the county GDP statistics, land use maps, night light brightness, and residential density, the spatial patterns of the GDP were obtained by the multi-factor weight distribution method. Based on the county demographic data, land use types, night light brightness, and residential density, the spatial patterns of the population density were obtained by the multi-factor weight distribution method. The meteorological datasets, including monthly precipitation and monthly temperature, were downloaded from the China Meteorological Administration (http://data.cma.cn, accessed on 25 April 2022). The quality of the meteorological datasets was controlled and the percentage of correct data approached 100%. The NDVI, meteorological datasets, GDP, population density, and land use type were resampled to match a spatial resolution of 1 km. The annual increment of artificial afforestation and the sown area of crops in the Pearl River Delta were obtained from the Statistical Yearbook of Guangdong Province (http://stats.gd.gov.cn/gdtjnj, accessed on 25 April 2022).

### 2.3. Slope Trend Analysis

A slope trend line analysis is a method to study the changing trend of a set of variables by performing a linear regression analysis on time-varying variables. In this paper, the univariate linear regression method was used to calculate the change trend of the vegetation NDVI value for each pixel and we analyzed the temporal changes of the vegetation NDVI in the study area [31]. The slope could be calculated as:

(1) $$\mathrm{slope}=\frac{n\times \sum_{i=1}^{n}{i\times f}_{\mathrm{vi}}-\sum_{i=1}^{n}i\sum_{i=1}^{n}f_{\mathrm{vi}}}{n\times \sum_{i=1}^{n}i^{2}-{\left(\sum_{i=1}^{n}i\right)}^{2}}$$

where the slope is the change trend slope of the vegetation NDVI, *n* is the number of years in the monitoring period, and $f_{\mathrm{vi}}$ is the vegetation NDVI value in the year *i*. If the slope > 0, it indicated that the vegetation NDVI showed an increasing trend; if the slope < 0, it indicated that the vegetation NDVI showed a downward trend [32].

### 2.4. Correlation Analysis

Climatic factors are one of the main factors affecting the vegetation NDVI. In this paper, we calculated the correlation coefficient between the vegetation NDVI and precipitation and temperature to analyze the correlation between the NDVI and precipitation and temperature [33,34]. The formula for calculating the correlation coefficient was:

(2) $$R_{\mathrm{xy}}=\frac{\sum_{i=1}^{n}[\left(x_{i}-\overset{-}{x}\right)\left(y_{i}-\overset{-}{y}\right)]}{\sqrt{\sum_{i=1}^{n}{\left(x_{i}-\overset{-}{x}\right)}^{2}\sum_{i=1}^{n}{\left(y_{i}-\overset{-}{y}\right)}^{2}}}$$

where $R_{\mathrm{xy}}$ is the correlation coefficient between the two variables, $x_{i}$ is the annual average NDVI of the year *i*, $y_{i}$ represents the annual average temperature or precipitation of the year *i*, and $\overset{-}{x}$ and $\overset{-}{y}$ represent the mean value of *x* and *y*, respectively. If $R_{\mathrm{xy}}$ > 0, it indicated that *x* and *y* were positively correlated and if $R_{\mathrm{xy}}$ < 0, it indicated that *x* and *y* were negatively correlated.

### 2.5. Residual Analysis

A residual analysis was proposed by Evans and Geerken (2004). A residual is the difference between the actual value and the predicted value. In this paper, we used the binary regression model to calculate the relationship between the NDVI and climatic factors to obtain the predicted value of the NDVI (the contribution of climatic factors to the NDVI, or NDVI_PRE_). The residual value (the contribution of human activities to the NDVI, or NDVI_HA_) was then obtained by subtracting the actual value of the NDVI from the predicted value. The NDVI_HA_ could be calculated as:

(3) $$\{\begin{array}{c} \begin{array}{c} {\mathrm{NDVI}}_{\mathrm{HA}}{=\mathrm{NDVI}}_{\mathrm{OBS}}-{\mathrm{NDVI}}_{\mathrm{PRE}}\\ {\mathrm{NDVI}}_{\mathrm{PRE}\;}=c+\mathrm{ax}+\mathrm{by}\\ a=\frac{R_{\mathrm{xz}}-R_{\mathrm{xy}}{\;\times \;R}_{\mathrm{yz}}}{1\;-{\;R}_{\mathrm{xy}}^{2}}\;\times \;\frac{U_{z}}{U_{x}} \end{array}\\ \;b=\frac{R_{\mathrm{yz}}-R_{\mathrm{xy}}{\;\times \;R}_{\mathrm{xz}}}{1-R_{\mathrm{xy}}^{2}}\;\times \;\frac{U_{z}}{U_{y}}\\ c=\bar{z}-a\;\times \;\bar{x}-b\;\times \;\bar{y} \end{array}$$

where ${\mathrm{NDVI}}_{\mathrm{HA}}$ represents the NDVI contributed by human activities; ${\mathrm{NDVI}}_{\mathrm{OBS}}$ represents the actual value of the NDVI; ${\mathrm{NDVI}}_{\mathrm{PRE}}$ represents the predicted value of the NDVI; *z* is the value of the NDVI; *x* and *y* are precipitation and temperature, respectively; and *R_xy_*, *R_xz_*, and *R_yz_* represent the correlation coefficients between precipitation and temperature, precipitation and the NDVI, and temperature and the NDVI, respectively. *U_x_*, *U_y_*, and *U_z_* represent the variances in precipitation, temperature, and the NDVI, respectively; and $\overset{-}{x}$, $\overset{-}{y}$, and $\overset{-}{z}$ represent the means of precipitation, temperature, and the NDVI, respectively. If ${\mathrm{NDVI}}_{\mathrm{HA}}$ > 0, it indicated that human activities had a positive impact on the NDVI. If ${\mathrm{NDVI}}_{\mathrm{HA}}$ < 0, it indicated that human activities had a negative impact on the NDVI. If ${\mathrm{NDVI}}_{\mathrm{HA}}$ = 0, it indicated that human activities had little effect on the NDVI [35].

## 3. Results

### 3.1. NDVI Interannual Variability

**Figure 2:**
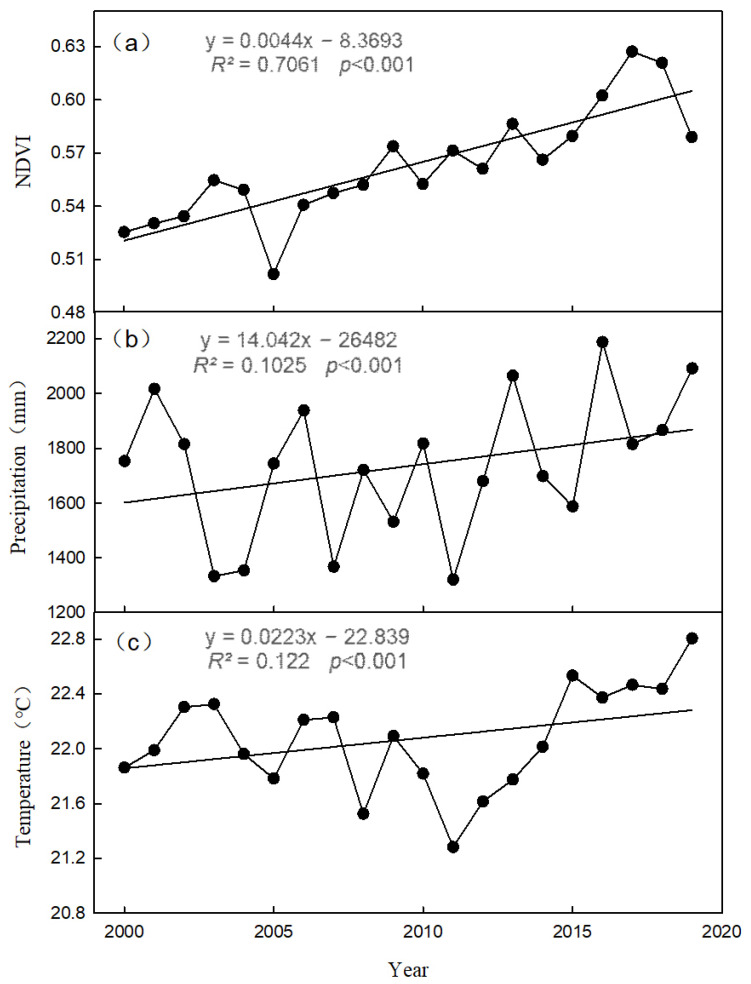
Temporal variation in (**a**) NDVI, (**b**) precipitation, and (**c**) temperature.

The vegetation NDVI in the Pearl River Delta showed a significant increasing trend with a rate of 0.0044 (*R*^2^ = 0.7061, *p* < 0.0001) during the period 2000–2019 (see Figure 2a), increasing from 0.53 in 2000 to 0.58 in 2019. The minimum value of the vegetation NDVI appeared in 2005 with a value of 0.50; the maximum value appeared in 2017 with a value of 0.63. From the data of the past 20 years, the vegetation NDVI in the Pearl River Delta showed a positive correlation with temperature and precipitation with correlation coefficients of 0.40 and 0.21, respectively; the correlation between temperature and the NDVI was stronger. The vegetation NDVI in the Pearl River Delta significantly decreased during 2004–2005; the reason for this was that the temperature significantly decreased in those years (see Figure 2c). The continuous decrease in the afforestation area caused the NDVI to decrease in 2018–2019.

### 3.2. Spatial Pattern of the NDVI

**Figure 3:**
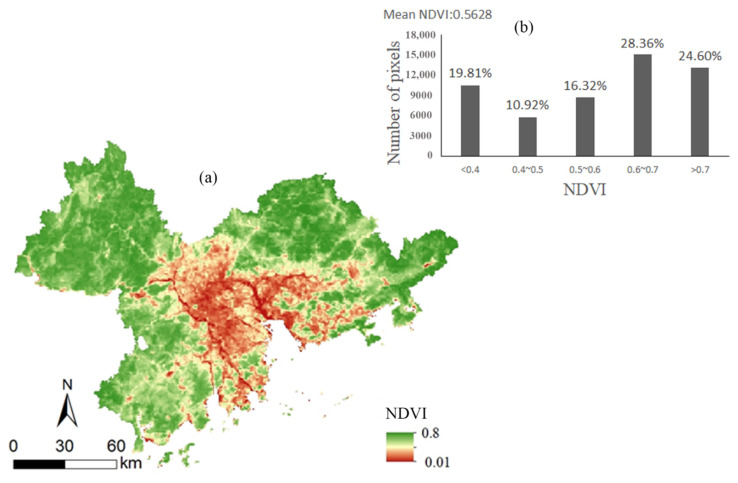
(**a**) Spatial pattern of average NDVI and (**b**) the ratio of pixels in the NDVI.

Figure 3a shows that the spatial pattern of the vegetation NDVI in the Pearl River Delta significantly varied. The high-value area of the NDVI (NDVI > 0.7) accounted for 24.6% of the study area (see Figure 3b) and was mainly distributed in Zhaoqing, northeastern Guangzhou, Huizhou, and Jiangmen. The land use types in those areas was mainly forest and cropland with a high vegetation coverage. The low-value area of the NDVI (NDVI < 0.4) accounted for 19.81% of the study area (see Figure 3b) and was distributed in central and eastern Foshan, northwestern Zhongshan, central Zhuhai, southern Guangzhou, Dongguan, and central and western Shenzhen. The land use type in those areas was mainly urban land with a low vegetation coverage.

### 3.3. Trend Analysis in the NDVI

**Figure 4:**
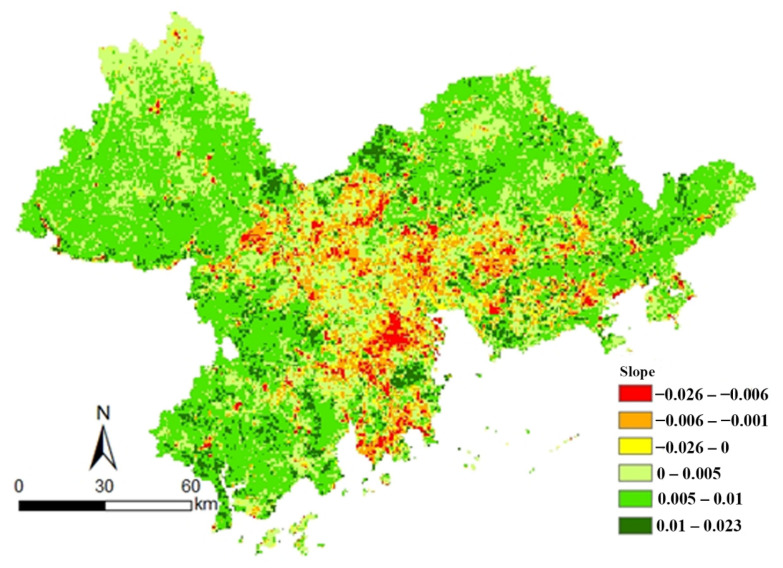
The trend of NDVI from 2000 to 2019.

The trend of the vegetation NDVI in the Pearl River Delta from 2000 to 2019 was calculated by a slope trend line analysis (see Figure 4). Most of the vegetation NDVI in the study area showed an increasing trend and the degraded areas were distributed in the center of the Pearl River Delta. The NDVI improvement area accounted for 84% of the total study area and was mainly distributed in the northwest of Zhaoqing and northern Huizhou. The trend of improvement was particularly significant in Jiangmen, southeastern Zhongshan, northern Guangzhou, eastern Huizhou, and southern Shenzhen. The NDVI-decreased area accounted for 16% of the total study area and was mainly in central Foshan, southern Guangzhou, northern Zhongshan, and central Dongguan. The decreasing trend was particularly significant in eastern Zhaoqing, northern Foshan, western Guangzhou, northern Zhongshan, southwestern Zhuhai, and west and east of Dongguan. According to the land use maps during the period 2000–2019, the land use types in the regions where the NDVI had increased in the Pearl River Delta had changed from land use types with a lower NDVI (such as urban land and river) to land use types with a higher NDVI (such as grassland, cropland, and forest land) and the area of those regions covered about 10.05% of the NDVI improvement area. There was 29.95% of NDVI-degraded regions where the land use types with a higher NDVI were changed to types with a lower NDVI. Therefore, land use change was the main cause of the NDVI variation.

### 3.4. Impact of Climatic Factors on the NDVI

**Figure 5:**
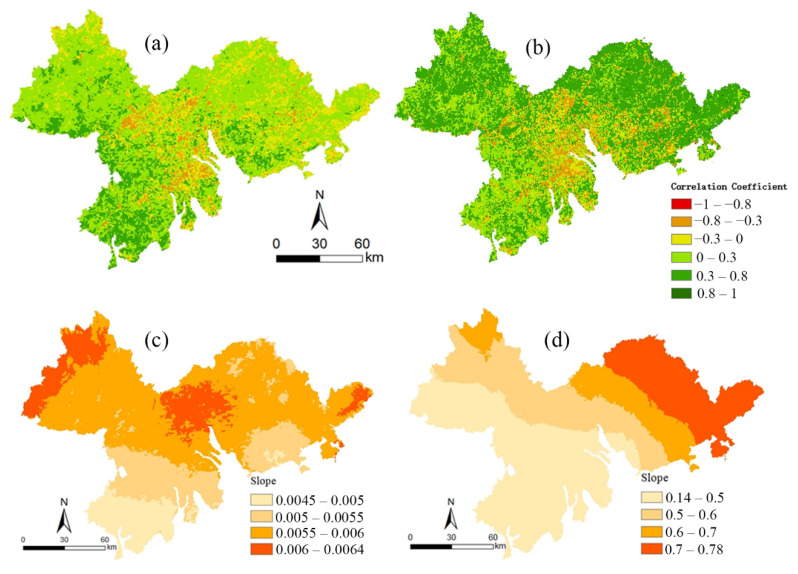
The spatial patterns of correlation coefficient (*R*) between NDVI and (**a**) precipitation and (**b**) temperature. The trends of (**c**) precipitation and (**d**) temperature from 2000 to 2019.

The correlation coefficient between the NDVI and temperature and the correlation coefficient between the NDVI and precipitation in the Pearl River Delta from 2000 to 2019 are shown in Figure 5. The correlation coefficient between the NDVI and precipitation was between −0.69~0.70, with an average value of 0.10. There was only 16.53% of the study area where the correlation coefficient between the NDVI and temperature was larger than or equal to 0.3 (see Figure 5a and Table 1). The correlation coefficient between the NDVI and temperature was between −0.84707 and 0.99997, with a mean value of 0.23261. There was 50.84% of the study area where the correlation coefficient between the NDVI and temperature was larger than or equal to 0.3 (see Figure 5b and Table 1).

As far as climatic factors are concerned, by comparing the magnitude of the correlation coefficient, it could be found that the correlation coefficient between the vegetation NDVI and temperature was generally higher than that of precipitation. The changes in temperature and precipitation both showed an increasing trend (see Figure 5c,d). The precipitation changes in the past 20 years were less obvious and the temperature showed an increasing trend. Therefore, temperature was the dominant climate factor affecting the change to the NDVI in the Pearl River Delta. This indicated that an appropriate increase in temperature could provide the heat supply required for vegetation growth and promote the growth of vegetation in the region. Compared with temperature, the correlation coefficient between the vegetation NDVI and precipitation was generally lower. This was consistent with the findings of He, Q.J. (2019). It could be seen that excessive precipitation could also inhibit the growth of vegetation.

### 3.5. NDVI_HA_ Interannual Variability

**Figure 6:**
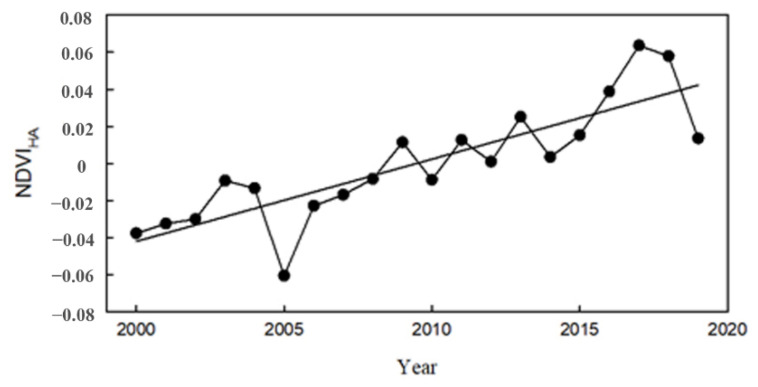
Temporal variation in NDVI_HA_.

In the past 20 years, the NDVI driven by human activities (NDVI_HA_) in the Pearl River Delta showed a fluctuating upward trend, increasing from −0.04 in 2000 to 0.01 in 2019; the growth rate was 0.0044 (*R*^2^ = 0.7062, *p* < 0.001) (see Figure 6). The impact of human activities on the vegetation NDVI increased and changed from negative to positive. Before 2009, the impact of human activities on the NDVI was mainly negative; after 2009, it was positive. The NDVI_HA_ in the Pearl River Delta significantly decreased from 2004 to 2005 and from 2018 to 2019, which was consistent with the fluctuation trend of the actual NDVI.

### 3.6. Trend Analysis in the NDVI_HA_

**Figure 7:**
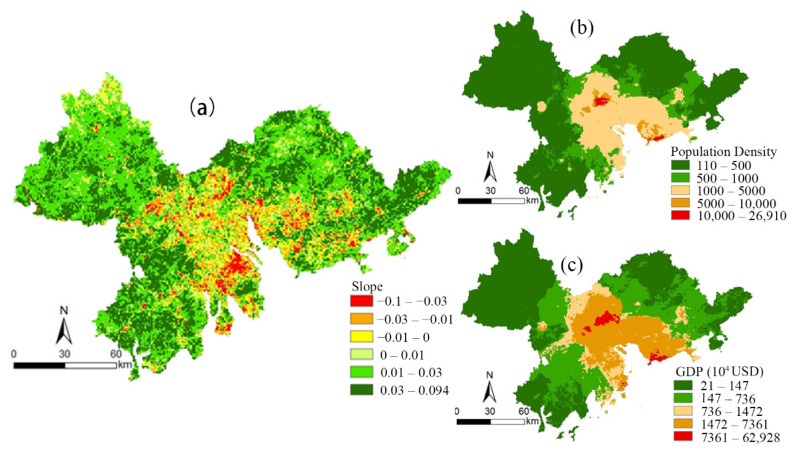
The spatial patterns of (**a**) trend of NDVI_HA_, (**b**) population density, and (**c**) GDP.

The spatial trend of the NDVI_HA_ in the Pearl River Delta from 2000 to 2019 was calculated by a slope trend line analysis (see Figure 7a). The area where the NDVI_HA_ showed an improvement trend was larger than the area where the NDVI_HA_ showed a downward trend. The regions where human activities had a positive impact on the NDVI accounted for 81.79% of the study area and these regions were mainly distributed in Zhaoqing, Huizhou, and Jiangmen. The regions where human activities had a negative impact on the NDVI accounted for 18.21% of the study area and these regions were mainly distributed in the urban cluster areas near the Pearl River Estuary. In those regions, including Guangzhou, Foshan, Zhongshan, Zhuhai, and Dongguan, the population density and GDP were higher than in other regions (see Figure 7b,c).

### 3.7. Impact of Human Activities on the NDVI_HA_

**Figure 8:**
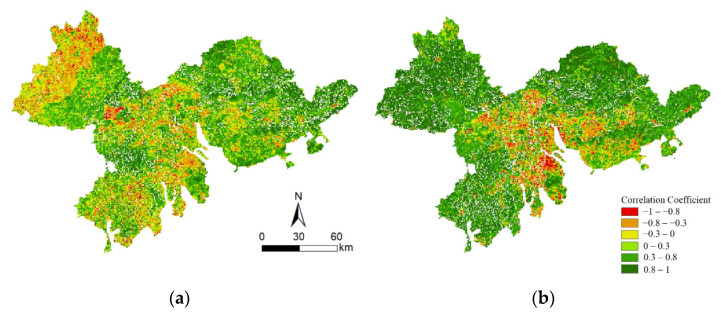
The spatial patterns of correlation coefficient (*R*) between NDVI_HA_ and (**a**) population density and (**b**) GDP.

The correlation coefficient between the NDVI_HA_ and population density and the correlation coefficient between the NDVI_HA_ and GDP in the Pearl River Delta from 2000 to 2019 are shown in Figure 8. The correlation coefficient between the NDVI_HA_ and population density was between −0.99986 and 0.99995, with an average value of 0.22581. There was 49.26% of the study area where the correlation coefficient between the NDVI_HA_ and population density was larger than or equal to 0.3 (see Figure 8a and Table 2). The correlation coefficient between the NDVI_HA_ and GDP was between −0.99959 and 0.99997, with an average value of 0.46444. There was 72.80% of the study area where the correlation coefficient between the NDVI_HA_ and GDP was larger than or equal to 0.3 (see Figure 8b and Table 2). The results showed that the correlation coefficient between the NDVI_HA_ and GDP was generally higher than that of population density, indicating that the GDP was the main human factor affecting the change to the NDVI_HA_ in the Pearl River Delta.

## 4. Discussion

**Figure 9:**
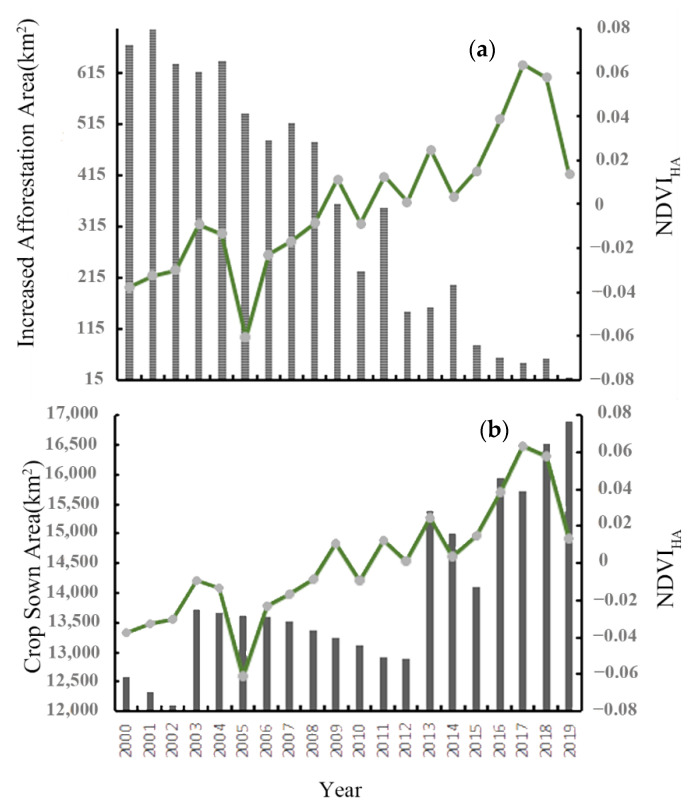
NDVI_HA_ with (**a**) increased afforestation area and (**b**) annual crop sown area.

In addition to climatic and socio-economic factors, policy factors also affected the changes in the NDVI. In 1998, the National Forestry and Grassland Administration of China launched a pilot project of natural forest protection in 12 provinces and autonomous regions, and in 1999 the “Grain for Green Project” was implemented to curb ecological deterioration, protect biodiversity, and promote the protection of natural forest resources. The latest research shows that from 2000 to 2017, the newly added green area of China accounted for about 1/4 of that of the world, ranking first in the contribution list [36]. In the Pearl River Delta region, the NDVI_HA_ showed a fluctuating growth trend with the growth of artificial afforestation (see Figure 9a) and showed a certain lag phenomenon. This was because changes to the state of the ecosystem structure require an adaptation cycle of several years, which reflects the cumulative ecological effects brought about by the afforestation policy [37]. However, with the rapid increase in the population density of the Pearl River Delta around 2010, the newly added afforestation area decreased rapidly after 2010 and the NDVI_HA_ significantly decreased from 2018 to 2019.

The sown area of crops is the area of land where crops are actually sown or transplanted and was calculated as the area of land where crops actually survive at the end of each planting season. Therefore, changes in the sown area of crops affect the NDVI in cropland. In the Pearl River Delta, the sown area of crops showed a fluctuating growth trend over the 20 years (see Figure 9b). The sown area of crops was low before 2013. However, in the context of the increasing demand for food due to population growth, food security has become a social issue of widespread concern [38]. Therefore, the sown area of crops increased rapidly from 2013, resulting in an increasing trend of the NDVI_HA_.

Human-dominated land use change is also an important driver of NDVI_HA_ changes [39]. According to the land use type maps from 2000 and 2020, the increase in urban land in the past 20 years was derived from the transformation of 2822 km^2^ of cropland, 1419 km^2^ of forest land, and 943 km^2^ of river, reflecting the large-scale occupation of cropland and forest land in the process of urbanization [30]. Land reclamation from the sea is a way to increase urban land and reduce damage to existing forest land, grassland and cropland. As the Pearl River Delta is located in a coastal area, it is an area where many rivers enter the sea and there are many suitable bays. In addition, estuaries and bays are often developed and linked together in coastal areas and have natural advantages such as in-depth land and a stable hydrological environment, which are convenient for development and utilization [40]. In order to solve the contradiction between vegetation cover and human activities, there has been 1.045 km^2^ of land reclamation in the Pearl River Delta to avoid destroying the existing woodland and grassland. These factors may also have caused the NDVI_HA_ to increase during the 2000–2019 period.

All in all, it is recommended that a focus is placed on strengthening the overall coordination of urban space, green space system, and ecological space in the Pearl River Delta region. In addition, core areas of ecological restoration are mainly concentrated in the Pearl River Delta. In the core areas of the region (Guangzhou, Foshan, Zhongshan, Zhuhai, and Dongguan), reasonable human activities need to consider the ecological security of the region. To achieve the organic combination of ecological and economic benefits, we should strengthen the implementation of artificial afforestation policies and build “green cities”.

## 5. Conclusions

In this paper, we used a residual analysis to analyze the variation in the NDVI and its driving factors in the Pearl River Delta region. The results showed that:

(1) From 2000 to 2019, the vegetation NDVI in the Pearl River Delta generally showed a slow upward trend and the vegetation coverage in most areas of the Pearl River Delta showed an improvement trend.
(2) Temperature and precipitation were the main climatic factors affecting the changes of the NDVI in the Pearl River Delta and the impact of temperature on the NDVI was greater than that of precipitation.
(3) In the past 20 years, the NDVI_HA_ driven by human activities has generally shown an upward trend and human activities have changed from a negative hindering effect on the rise of the NDVI to a positive promoting effect. The impact of the GDP on the NDVI_HA_ was greater than that of population density.
(4) Policy factors were also an important driving factor affecting changes to the NDVI in the Pearl River Delta. The “Grain for Green Project” and increasing sown areas of crops caused the NDVI increase; the occupation of forest land and grassland by urban land also caused an NDVI decrease.

## Figures and Tables

**Table 1:** Correlation coefficient (*R*) and proportion of the area in the Pearl River Delta between the NDVI changes and natural driving factors (precipitation and temperature).

*R* between NDVI and Precipitation	Proportion	*R* between NDVI and Temperature	Proportion
*R* ≤ −0.8	0%	*R* ≤ −0.8	0.01%
−0.8 < *R* ≤ −0.3	5.15%	−0.8 < *R* ≤ −0.3	8.51%
−0.3 < *R* < 0.3	78.32%	0.3 < *R* < 0.3	40.63%
0.3 ≤ *R* < 0.8	16.53%	0.3 ≤ *R* < 0.8	50.46%
*R* ≥ 0.8	0%	*R* ≥ 0.8	0.38%

**Table 2:** Correlation coefficient (*R*) and proportion of the area in the Pearl River Delta between the NDVI_HA_ changes and human driving factors.

*R* between NDVI_HA_ and Population Density	Proportion	*R* between NDVI_HA_ and GDP	Proportion
*R* ≤ −0.8	2.15%	*R* ≤ −0.8	2.81%
−0.8 < *R* ≤ −0.3	15.22%	−0.8 < *R* ≤ −0.3	10.13%
−0.3 < *R* < 0.3	33.37%	−0.3 < *R* < 0.3	10.13%
0.3 ≤ *R* < 0.8	36.16%	0.3 ≤ *R* < 0.8	39.60%
*R* ≥ 0.8	13.1%	*R* ≥ 0.8	33.20%

## Data Availability

Not applicable.
